# Targeting ferroptosis: New perspectives of Chinese herbal medicine in the treatment of diabetes and its complications

**DOI:** 10.1016/j.heliyon.2023.e22250

**Published:** 2023-11-11

**Authors:** Cuiping Liu, Wuxi Wang, Junling Gu

**Affiliations:** a Department of Endocrinology, The Second People's Hospital of Yibin City-West China Yibin Hospital, Sichuan University, Yibin, Sichuan, PR China; b Clinical Research and Translation Center, Second People's Hospital of Yibin City-West China Yibin Hospital, Sichuan University, Yibin, Sichuan, PR China; c Community Health Service Center of Tongyuanju, Chongqing, PR China

**Keywords:** Ferroptosis, Chinese herbal medicine, Natural compounds, Diabetes, Diabetic complications

## Abstract

Ferroptosis is a non-apoptotic mode of cell death. A large number of studies have confirmed that ferroptosis plays a vital role in the occurrence and development of diabetes and diabetic complications. Previous studies have found that Chinese herbal medicines have very promising results in the prevention and treatment of diabetes and diabetic complications, and some of these herbs or herbal natural compounds may act via the inhibition of ferroptosis. In this review, we summarized the relationship between ferroptosis and diabetes and diabetic complications, and discussed its molecular mechanisms. We also reviewed the published studies of herbal medicines or herbal natural compounds that improved diabetes or diabetic complications via the ferroptosis pathway. In addition, we are trying to provide new insights for better treatment of diabetes and diabetic complications with Chinese herbal medicine and its herbal compounds.

## 1 Introduction

The prevalence of diabetes is gradually increasing. According to the International Diabetes Federation, the global diabetes prevalence in 20–79-year-olds in 2021 was estimated to be 10.5 % (536.6 million people), rising to 12.2 % (783.2 million) in 2045 [1]. Diabetes and diabetic complications significantly affect the patient's quality of life and cause a huge economic burden to families and society. However, its prevention and treatment are still difficult due to its complex pathogenesis. Traditional Chinese medicine has recorded knowledge of diabetes for over 2000 years [2]. Numerous studies have confirmed that Chinese herbal medicines or their natural compounds can significantly improve glycemic control and clinical indicators in diabetic patients, and effectively delay and improve the course of diabetes and its complications [3]. However, its mechanism is entirely unclear, and it may be related to inhibiting oxidative stress, increasing anti-inflammatory signaling pathways, regulating gut microbiota [4], and regulating various types of programmed cell death [5,6] (see ).

Ferroptosis, a non-apoptotic mode of cell death, was first officially proposed by Dixon et al. [7]. It is characterized by an increase in iron-dependent lipid peroxides and reactive oxygen species (ROS), which is morphologically manifested as mitochondrial shrinkage, increased mitochondrial membrane density, and reduced or disappeared mitochondrial cristae [8]. Ferroptosis is regulated by multiple metabolic pathways, including iron metabolism, glutathione peroxidase 4 (GPX4), and lipid metabolism [9]. The various regulatory mechanisms are interrelated. Previous studies have shown that ferroptosis is associated with various physiological and pathological processes such as tumors, infectious diseases, neurodegeneration, tissue damage, autoimmune diseases [10], diabetes, and osteoporosis [11]. In addition, recent studies have reported that Chinese herbal medicines or natural compounds of herbal medicines may improve diabetes and diabetic complications by regulating pathways of ferroptosis. This review summarized the relationship between ferroptosis and diabetes and its complications, and reviewed the published studies of herbal medicines or herbal natural compounds that improved diabetes or its complications via the ferroptosis pathway. This may provide important ideas for studying new target drugs for the prevention and treatment of diabetes and its complications.

## 2 Ferroptosis and diabetes and diabetic complications

### 2.1 Ferroptosis and diabetes

Ferroptosis plays an important role in the pathogenesis of diabetes and its complications. If the iron content in the body is excessively increased for various reasons, it is possible to cause severe damage to pancreatic cells through excessive oxidative stress, and the ability of the liver to utilize insulin and gluconeogenesis is weakened, leading to the occurrence and development of type 2 diabetes mellitus (T2DM) [12]. Studies have shown that iron overload, the key initiating factor of ferroptosis, will aggravate insulin resistance in the absence of inflammation in diabetic mice [13]. Ferroptosis has been shown to occur in the pancreas of mice with T2DM and high glucose (HG) induced INS-1 cells [14]. Zhang, Shanshan et al. found that the up-regulation of miR-144–3p suppressed USP22/Sirtuin 1 (Sirt1) to induce ferroptosis, which caused pancreatic β cells dysfunction, thereby promoting T2DM development [14]. The main pathological hallmark of diabetes is the loss of functional β-cells [15]. Ferrostatin 1 (Fer-1), ferroptosis inhibitor, protected pancreatic islets from streptozotocin (STZ) induced injury in diabetic in *vivo* model [15]. Thus, ferroptosis may play an important role in the mechanism of STZ-induced pancreatic damage leading to diabetes, but the in-depth mechanism is still unclear.

### 2.2 Ferroptosis and diabetic nephropathy

Diabetic nephropathy(DN) is a serious microvascular complication of diabetes mellitus and has been recognized as the leading cause of end-stage renal disease [16,17]. The kidney plays an important role in the metabolism of iron [18]. Oxidative stress weakens antioxidant capacity, and iron overload is an important pathogenesis of DN [19]. Disturbances in cellular and systemic iron balance are recognized as causes and consequences of kidney injury [18]. Bioinformatics analysis of over 250 microarray datasets has implicated that ferroptosis is associated with renal tubular cell death in patients with diabetes [20]. The results of weighted gene co-expression network analysis and enrichment pathway analysis have indicated that ferroptosis has significantly occurred in the advanced DN group [21]. Moreover, a study has found that compared with healthy controls, patients with DN have higher levels of serum ferritin, lactate dehydrogenase (LDH), ROS and malondialdehyde (MDA), and ferroptosis-related factors are dysregulated, including Acyl-CoA synthetase long-chain family member 4 (ACSL4), prostaglandin-endoperoxide synthase 2 (PTGS2), NADPH oxidase 1 (NOX1), and GPX4 [22]. Ferroptosis is associated with the elevation of ACSL4, PTGS2, and NOX1, and the reduction of GPX4 [23]. These studies suggest that ferroptosis is associated with the development of DN [16].

**Fig. 1:**
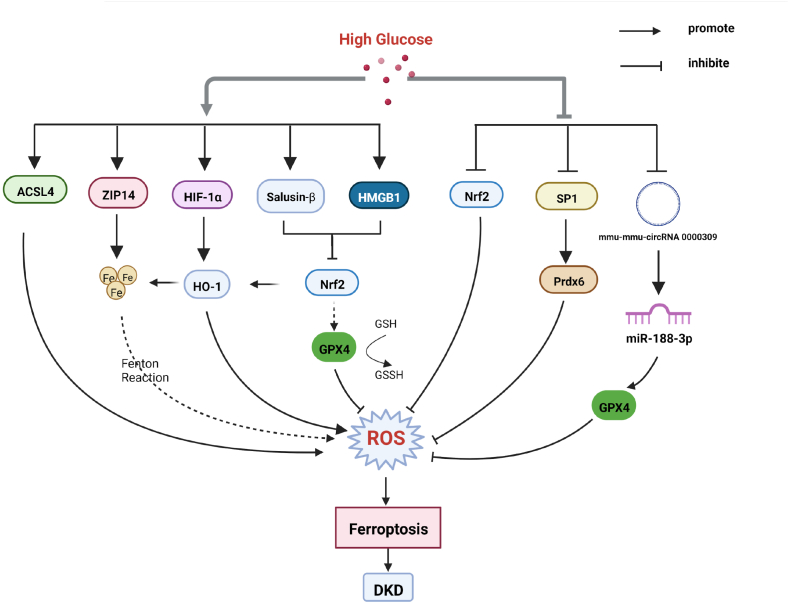
High glucose induces ferroptosis by various pathways, participating in the occurrence of diabetes nephropathy. Partial abbreviation: ACSL4: Acyl-CoA synthetase long-chain family member 4; ZIP14: ZRT/IRT-like protein 14; HIF-1α：hypoxia-inducible factor-1α; HMGB1: High-mobility group box-1; Nrf2:nuclear factor E2-related factor 2; SP1:specificity protein 1; GPX4:glutathione peroxidase 4. ROS: reactive oxygen species.

ACSL4 catalyzes the eventual production of harmful lipid peroxidation products PE-AA-OOH and PE-ADA-OOH, which when accumulated in excess lead to ferroptosis [24]. The ACSL4 inhibitor rosiglitazone could improve renal function and decrease lipid peroxidation products and iron content in DN mice, and these effects are associated with reduced ferroptosis [25]. Thus, ACSL4 may be a new therapeutic target for DN. High-mobility group box-1 (HMGB1) is a DNA-bound non-histone protein extracellularly thought to be a pro-inflammatory mediator of human disease [26]. A study has found that inhibition of HMGB1 restores HG-induced mesangial cell proliferation, decreases ROS production, and reverses ferroptosis [22]. Moreover, HMGB1 may regulate glucose-induced ferroptosis in mesangial cells via the nuclear factor E2-related factor 2 (Nrf2) pathway, including its downstream targets heme oxygenase-1 (HO-1), NQO-1, GCLC, and GCLM [22]. A study has found that umbelliferone significantly ameliorates renal pathological damage and ROS accumulation in db/db mice, down-regulates ACSL4, and up-regulates the expressions of GPX4, Nrf2, and HO-1 [27]. Furthermore, the knockdown of Nrf2 blocks the inhibitory effect of umbelliferone on high glucose-induced ferroptosis in renal tubular cells [27], in which the Nrf2/HO-1 pathway may play a role in DN. In another study, specific knockdown of Nrf2 increases the sensitivity of HK-2 cells to ferroptosis under high glucose conditions, whereas upregulation of Nrf2 improved ferroptosis in diabetic HK-2 cells [28]. As a bioactive peptide, salusin-β is abundantly expressed in the kidneys. High glucose could upregulate the expression of salusin-β, which inactivated Nrf-2 signaling, ultimately leading to elevated levels of ferroptosis in HK-2 cells [29]. Thus, Nrf2 plays a key role in the ferroptosis pathway in diabetic nephropathy. But Xu et al. found that excessive activation of Nrf2 can produce a lot of fat and glycogen, leading to hepatic steatosis and glucose intolerance [30]. The regulatory balance point of Nrf2 deserves further study [31]. In addition, ferroptosis enhanced diabetic renal tubular injury via hypoxia-inducible factor (HIF)-1α/HO-1 pathway in db/db mice [32]. It can be seen that HO-1 also seems to have a dual role in ferroptosis, which is worthy of our further study. Targeting NADPH-mediated ROS release and ferroptosis accumulation is a novel therapeutic strategy to protect the kidney from septic injury in patients with obesity and T2DM [33]. In DN, podocytes are injured early in the disease course [34]. The specificity protein 1 (Sp1)-mediated upregulation of peroxiredoxin 6 (Prdx6) expression *in vitro* has been found to prevent podocyte injury in diabetic nephropathy by reducing oxidative stress and ferroptosis [35]. ZRT/IRT-like protein 14 (ZIP14) is a transporter that mediates the cellular uptake of iron, zinc, and manganese. The study has found that ZIP14 is involved in iron deposition and triggers ferroptosis in patients with DN [36]. N-acetylcysteine (NAC) alleviated ferroptosis in DN by maintaining mitochondrial redox homeostasis via activation of the SIRT3-superoxide dismutase 2 (SOD2)/Gpx4 pathway [37]. mmu_circRNA_0000309 is lowly expressed in podocytes of DN mice [38]. mmu_circRNA_0000309 sponges miR-188–3p, subsequently upregulates GPX4 expression, inactivating ferroptosis-depended mitochondrial function [38]. The relationship between ferroptosis and DN is shown in Fig. 1.

### 2.3 Ferroptosis and diabetic retinopathy

**Fig. 2:**
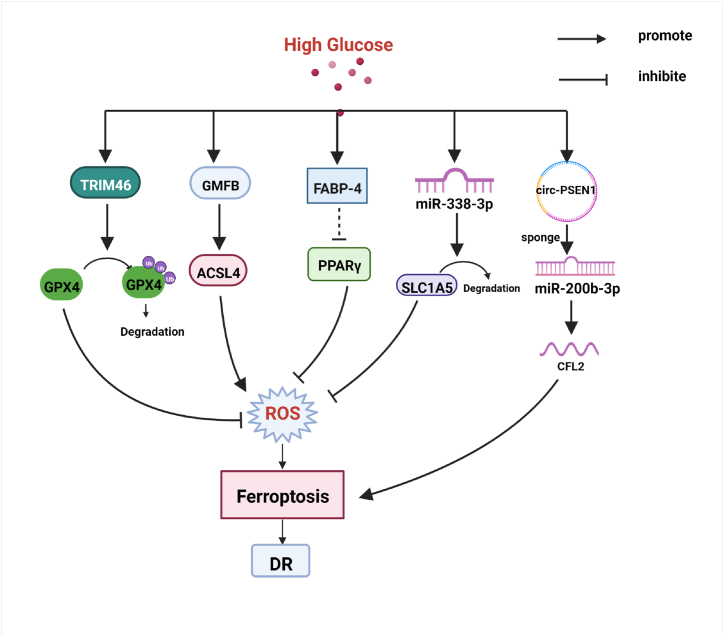
High glucose induces ferroptosis via various pathways, participating in the occurrence of diabetic retinopathy (DR). Partial abbreviation: TRIM46: a member of The E3 ubiquitin ligase family; GMFB: Glia maturation factor-β; ACSL4: Acyl-CoA synthetase long-chain family member 4; FABP-4: Fatty acid binding protein 4; PPARγ: Peroxisome proliferator-activated receptor γ; SLC1A5: Recombinant solute carrier family 1, member 5; CFL2: Cofilin-2; GPX4: glutathione peroxidase 4. ROS: reactive oxygen species.

Diabetic retinopathy (DR) is one of the leading causes of blindness in the world. It is important to find potential pathogenic mechanisms and therapeutic targets for timely intervention. The evidence supports that high glucose can promote ferroptosis in retinal pigment epithelial (RPE) cells [39]. Glia maturation factor-β (GMFB) induces ferroptosis by impairing chaperone-mediated autophagic degradation of ACSL4 in early diabetic retinopathy [40]. The upregulation of Thioredoxin-interacting protein (TXNIP) in RPE cells under high glucose and downregulation of antioxidant proteins will lead to the generation of oxidized GSSG and depletion of glutathione (GSH) [41]. Reduced GSH/GPX4 activity leading to iron accumulation and membrane lipid peroxidation is one of the mechanisms leading to ferroptosis [42]. TRIM46 is a gene located at chromosome 1q21 [43], which contributes to high glucose-induced ferroptosis and cell growth inhibition in human retinal capillary endothelial cells by facilitating GPX4 ubiquitination [44]. These studies have indicated that TRIM46 and GPX4 are the molecular targets of effective drugs for DR therapy. Moreover, microRNAs (miRNAs) are closely related to the development of various diseases, especially neoplastic diseases [45]. While recent data suggests that targeting related miRNAs, such as miR-338–3p, is a novel strategy to improve DR [46]. Mechanistically, HG can lead to recombinant solute carrier family 1, member 5 (SLC1A5) deletion in RPE cells by upregulating miR-338–3p, leading to oxidative stress-mediated ferroptosis and ultimately aggravating DR progression [47]. A study *in vitro* has demonstrated that the knockdown of circ-PSEN1 can mitigate ferroptosis of retinal pigment epithelial cell line-19 (ARPE19) cells induced by HG via the miR-200 b-3p/cofilin-2 axis [48]. Besides, the downregulation of fatty acid binding protein 4 (FABP4) alleviates lipid peroxidation and oxidative stress in DR retinal pigment epithelial cells by modulating Peroxisome PPARγ-mediated ferroptosis [49]. The relationship between ferroptosis and DR is shown in Fig. 2

### 2.4 Ferroptosis and diabetic cardiovascular and cerebrovascular complications

**Fig. 3:**
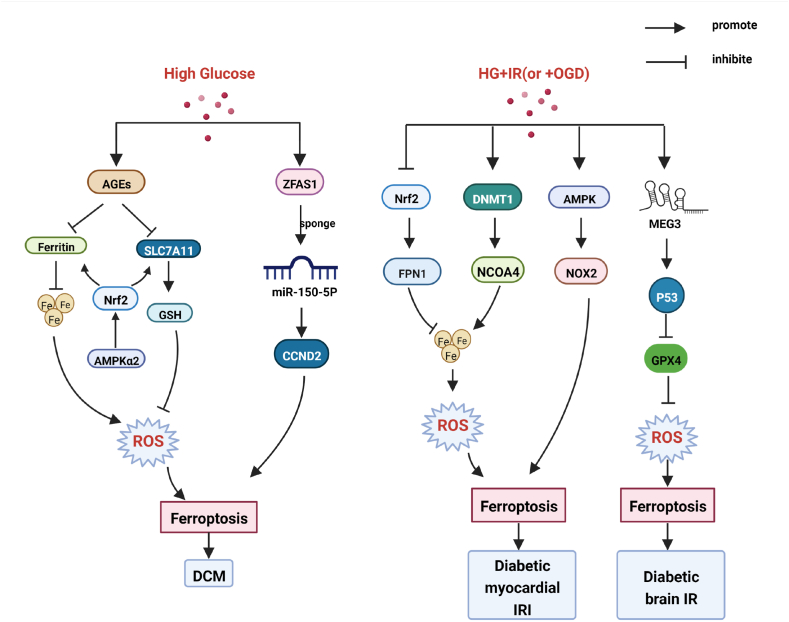
High glucose (HG) induces ferroptosis via various pathways, participating in the occurrence of diabetic cardiovascular and cerebrovascular complications. Partial abbreviation: AGEs: Advanced glycation end-products; SLC7A11: Solute carrier family 7 member 11; GSH: glutathione; AMPK: Adenosine 5′-monophosphate activated protein kinase; ZFAS1: Zinc finger antisense 1; CCND2: Cyclin D2; FPN1: Ferroportin1; DNMT: DNA methyltransferase; NCOA4: Nuclear receptor coactivator 4; NOX2: Nicotinamide adenine dinucleotide phosphate oxidase; IRI: ischemia/reperfusion injury.

Ferroptosis is associated with ischemia/reperfusion injury (IRI) and several other cardiac diseases as a significant form of cell death in cardiomyocytes [50]. Ferroptosis also plays an important role in diabetic cardiovascular and cerebrovascular diseases (see Fig. 3). Endothelial dysfunction, a hallmark of diabetes, is a key and initiating contributor to the pathogenesis of cardiovascular complications of diabetes. Ferroptosis is involved in endothelial dysfunction and p53-xCT-GSH axis activation plays a crucial role in endothelial cell ferroptosis and endothelial dysfunction [51]. Advanced glycation end-products (AGEs), an important pathogenic factor of DCM, were found to induce ferroptosis in engineered cardiac tissues [52]. Ferroptosis is essential for diabetic cardiomyopathy (DCM) and is prevented by sulforaphane via adenosine 5′-monophosphate activated protein kinase (AMPK)/Nrf-2 pathways [52]. Diabetes aggravates myocardial I/RI by generating Nox2-associated oxidative stress in an AMPK-dependent manner, leading to programmed cell death such as apoptosis, pyroptosis, and ferroptosis [53]. Nrf-2 controls the transcription of ferroportin1 (FPN1), the only mammalian protein associated with iron release [54]. Tian, H et al. have shown that iron homeostasis-related ferroptosis plays an important role in aggravating myocardial IRI in diabetic rats, and Nrf-2/FPN1 pathway-mediated iron homeostasis and ferroptosis may be a promising therapeutic target against myocardial IRI in diabetes [54]. Another study has found that inhibiting DNA methyltransferase 1 (DNMT-1) can alleviate ferroptosis through nuclear receptor coactivator 4 (NCOA4) mediated ferritinophagy during diabetes myocardial IRI [55]. The long non-coding RNA (lncRNA) zinc finger antisense 1(ZFAS1) acts as an endogenous RNA (ceRNA) to sponge miR‐150–5p and downregulates Cyclin D2 (CCND2) to promote cardiomyocyte ferroptosis and DCM development [56]. Therefore, ZFAS1 inhibition may be a novel target for the treatment and prevention of DCM. Besides, lncRNA Meg3 has been considered an important mediator in regulating ischemic stroke [57]. A study by Chen, C et al. has found that LncRNA Meg3 mediates ferroptosis induced by oxygen and glucose deprivation combined with hyperglycemia in rat brain microvascular endothelial cells, through modulating the p53/GPX4 axis [57].

### 2.5 Ferroptosis and diabetic osteoporosis

**Fig. 4:**
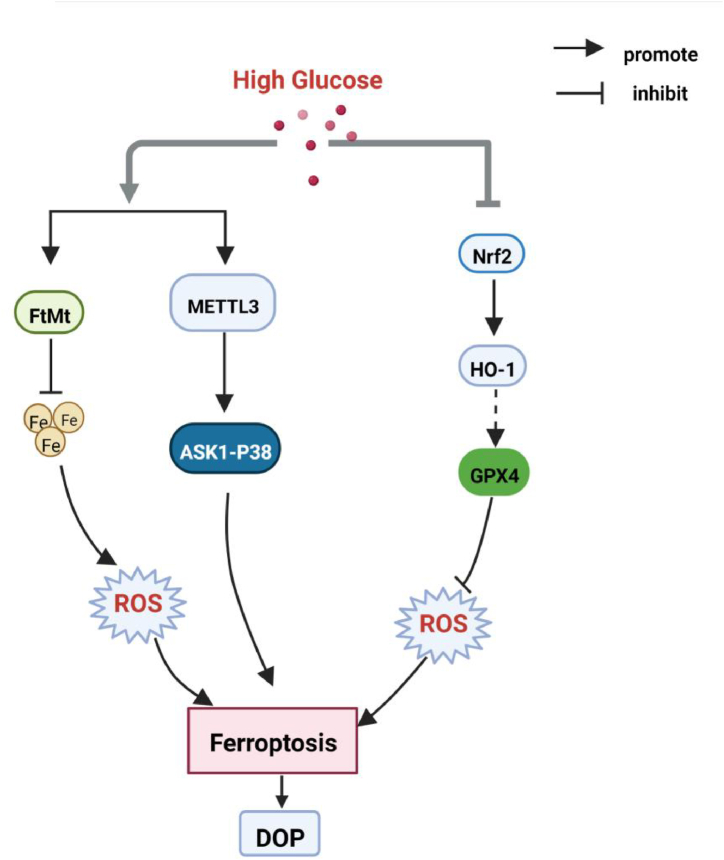
High glucose induces ferroptosis via various pathways, participating in the occurrence of diabetic osteoporosis (DOP). Partial abbreviation: FtMt: Mitochondrial ferritin; METTL3: methyltransferase-like 3; ASK1: Apoptosis signal-regulating kinase 1; HO-1: Heme oxygenase-1.

Diabetic Osteoporosis (DOP) is a systemic metabolic disease, a systemic bone disease characterized by low bone mass, destruction of bone microstructure, increased bone fragility, and susceptibility to fractures based on diabetes. A key pathogenic factor in DOP is loss of osteocyte viability. However, the mechanism of osteocyte death remains unclear. Some studies have indicated that abnormal iron metabolism increases the incidence of osteoporosis [58]. A study has found ferroptosis in the bone tissue of rats with type 2 diabetic osteoporosis [59]. Another study has detected that the diabetic microenvironment significantly enhances osteocyte ferroptosis *in vitro*, as manifested by massive lipid peroxidation, iron overload, and abnormal activation of the ferroptosis pathway [60]. Mitochondrial ferritin (FtMt) is a protein that stores iron ions and intercepts toxic ferrous ions in the mitochondria of cells [61]. This study further proves that FtMt overexpression reduces ferroptosis in osteoblasts under high glucose conditions, while silencing FtMt induces mitochondrial phagocytosis through the ROS/PINK1/Parkin pathway [59]. A recent study has found that high glucose and high fat-induced ferroptosis in osteoblasts may be the main cause of osteoporosis in DM by activating the methyltransferase-like 3 (METTL3)/Apoptosis signal-regulating kinase 1 (ASK1)-p38 signaling pathway [62]. One study has proved that targeting ferroptosis or HO-1 could efficiently rescue osteocyte ferroptosis in DOP by disrupting the vicious cycle of lipid peroxidation and HO-1 activation, eventually ameliorating trabecular deterioration [60]. In addition, another study has indicated that melatonin can inhibit the ferroptosis of osteoblasts by activating the Nrf2/HO-1 signaling pathway to improve bone microstructure *in vivo* and *in vitro* [63]. The activation of the Nrf2/HO-1 signal transduction pathway increased GPX4 activity and inhibited the ferroptosis of HG-induced osteoblasts [63]. The role of Nrf2/HO-1 in regulating DOP ferroptosis is controversial and requires further investigation. Altogether, these studies suggest that inhibition of ferroptosis in osteoblasts may provide a potential therapeutic strategy for diabetic osteoporosis (see Fig. 4).

### 2.6 Ferroptosis and diabetic cognitive impairment

Cognitive dysfunction is a growing complication of type 2 diabetes. Ferroptosis has been reported to be a major causative factor in diabetes-associated cognitive dysfunction [64]. An, Ji-Ren and colleagues have first demonstrated ferroptosis in the hippocampus in a T2DM model, which may play a role in diabetic cognitive impairment [65]. Inhibition of ferroptosis in hippocampal neurons improves neuronal damage in type 2 diabetes [66], but the specific molecular pathway remains unclear. A study has found that the overexpression of caveolin-1 may attenuate diabetes-associated cognitive dysfunction by modulating neuronal ferroptosis-mediated mitochondrial homeostasis [67]. Moreover, Liraglutide is found to prevent hippocampal ferroptosis by increasing the expression of GPX4 and solute carrier family 7 member 11 (SLC7A11) and inhibiting the overexpression of ACSL4, thereby restoring cognitive function [65]. As we all know SLC40A1 encodes a ferroportin (FPN). Hao, L et al. found that ferroptosis is associated with diabetic cognitive dysfunction and SLC40A1 mediates ferroptosis in type 1 diabetes [64]. Another study has shown that Rabbit Mixture could improve the cognitive function of diabetic cognitive dysfunction rats by regulating glutathione and L-cysteine, or it may be related to the ferroptosis pathway [68]. Therefore, research on drugs or agents targeting ferroptosis may provide new treatment strategies for patients with diabetes.

## 3 Chinese herbal medicine or herbal natural compounds in improving diabetes and its complications via ferroptosis pathway

Chinese herbal medicines and herbal natural compounds have achieved remarkable curative effects in lowering blood glucose and improving diabetic complications. Studies have shown that some herbal natural compounds such as berberine, pueraria, quercetin, mulberry leaves [6], artemisinin [69], astragalus, etc. have anti-diabetic effects, as shown in Table 1. Animal and human studies illustrate the potential benefit of mulberry leaf extract (MLE) in T2DM. A clinical trial has shown that mulberry leaf extract may be a useful complementary mealtime glucose option for patients with T2DM [70]. Eating foods rich in epicatechin and quercetin can effectively reduce FPG, total cholesterol, LDL-cholesterol, and total triglycerides in patients with metabolic syndrome [71]. Plant extracts rich in flavonoids, phenols, and quercetin reduced the levels of FPG, 2hPPG, HbA1c, TC, LDL-C, and triglycerides in T2DM, but increased the level of HDL-C (P < 0.05) [72]. In clinical practice, puerarin can reduce blood viscosity, improve microcirculation, and play a positive therapeutic role in diabetic retinopathy [73]. Berberine combined with fenugreek can decrease fasting blood sugar, fasting insulin, HbA1c and improve insulin resistance [74]. Besides, berberine combined with probiotics significantly reduces postprandial hyperlipidemia and CVD risk in patients with diabetes [75]. Resveratrol andδ-tocotrienol mixture supplementations improved cardiometabolic risk factors and biomarkers of inflammation and oxidative stress in patients with Metabolic syndrome [76]. The specific mechanism is not yet very clear. In recent years, ferroptosis, as a new type of cell death, has attracted attention in the field of traditional Chinese medicine for the treatment of diabetes. At present, there are some related studies, and the current research progress on Chinese herbal medicine or herbal natural compounds and the ferroptosis pathway in diabetes will be reviewed, as shown in Table 2.

**Table 1:** Clinical trials of Chinese herbal medicines or herbal natural compounds in improving diabetes and its complications.

Study	Herbs or herbal compounds	participants	Intervention time	clinical trial	Results
Riche, Daniel M et al. [70]	Mulberry leaf extract (MLE)	T2DM （n = 24）	3 months.	Randomized, double-blind, placebo controlled	Reduce postprandial blood glucose
Leyva-Soto, Aldo et al. [71]	The mixture of epicatechin and quercetin (1:1)	Patients with metabolic syndrome （n = 156）	3 months	A randomized placebo-controlled study	Reduce glucose and improve biochemical parameters related to metabolic syndrome.
Kianbakht, Saeed et al. [72]	plant extract(containsflavonoid，phenolic and quercetin)	Hypercholesterolemic type 2 diabetic patients. (n = 50)	2 months.	A randomized placebo-controlled study	The extract lowered FG, 2hPPG, HbA1c, TC, LDL-C, and triglyceride levels, but increased HDL-C levels compared to the placebo at the endpoint (*P* < 0.05).
Ren, P et al. [73]	Puerarin	DR （n = 15）	6 weeks.	A randomized placebo-controlled study	Reduces blood viscosity, improves microcirculation, and plays a positive therapeutic role in diabetic retinopathy.
Nematollahi, Shima et al. [74]	Berberine combined with fenugreek	T2DM (n = 50)	12 weeks.	A randomized controlled clinical trial	Improve cardio-metabolic status in patients with diabetes and support the anti-diabetic and anti-inflammatory role of herbs in the improvement of quality of life.
Wang, Shujie [75]	Berberine combined with probiotics	T2DM (n = 365)	12 weeks.	A random, placebo-controlled, and multicenter clinical trial	Significantly reduce postprandial hyperlipidemia and CVD risk in patients with diabetes.
Fatima, Safia et al. [76]	δ-tocotrienol and resveratrol mixture	Patients with metabolic syndrome (n = 41）	24 weeks	A randomized placebo-controlled study	Reduce the waist circumference, blood pressure, and FPG.

Partial abbreviation: T2DM: type 2 diabetes mellitus; DR: diabetic retinopathy; FG：fasting glucose; 2hPPG: 2-h post-prandial blood glucose; HbA1c: the hemoglobin A1c; TC: total cholesterol; LDL-C: low-density lipoprotein cholesterol; HDL-C: high-density lipoprotein cholesterol; CVD: cardiovascular disease.

**Table 2:** Summary of Chinese herbal medicines or herbal natural compounds in improving diabetes and its complications via the ferroptosis pathway.

Chinese herbal medicines or herbal natural compounds	Source	Experimentalmodels	Possible mechanisms	Type of disease	Outcomes
GBE-5 [77]	Ginseng berry	HepG2 cells (the KEGG metabolic pathway enrichment analysis)	ferroptosis	T2DM	Hypoglycemic effect.
Cryptochlorogenic acid [78]	Mulberry leaf	INS-1 cells; SD rats	Inhibit ferroptosis via the activation of XC−/GPX4/Nrf2 and inhibition of NCOA4.	T2DM	Improve the blood glucose level and islet injury.
Quercetin [79]	Flavonoids	C57BL/6J mice	Inhibit ferroptosis by increasing GSH activity, SOD activity, and GPX4 expression, and decreasing xCT, MDA expression and iron concentrations.	T2DM	Alleviate ferroptosis of pancreatic β Cells.
Puerarin [80]	Radix puerariae	HBZY-1 cells SD rat	Decrease cellular LDH and Lipid ROS Downregulate the expression of ferroptosis inducer ACSL4. Elevate the level of GPX4 level.	DN	Attenuate excessive extracellular matrix accumulation.
Platycodin D [81]	Dry root of Platycodon grandiflorm	HK-2 cells	Inhibit HG-induced ferroptosis. Downregulate ACSL4 and TFR1 expression Up-regulate GPX4, FTH-1 and SLC7A11 expression.	DN	Reverse the effects of HG condition on cell death.
Glabridin [82]	The main active component in licorice.	NRK-52E cells SD rats	Represse ferroptosis by increasing SOD and GSH activity, and GPX4, SLC7A11, and SLC3A2 expression, and decreasing MDA and iron concentrations, and TFR1 expression.	DN	Ameliorate the renal pathologicalchanges and the renal function Reduce FBG, HOMA-β, and HOMA-insulin index.
Umbelliferone [27]	widely exists in Umbelliferae plants	db/db mice	Attenuate HG-induced ferroptosis by activating the Nrf2/HO-1 pathway.	DN	Improve the renal pathological damage and ROS accumulation of db/db mice.
Calycosin [83]	Astragali Radix	HK-2 db/db mice	Inhibit HG-induced decrease in glutathione and GPX4 expression. Inhibit the increase of LDH, MDA, lipid ROS and NCOA4.	DN	Protective effect on DN may be through inhibition of ferroptosis pathway.
Sennoside A [84]	Rhei Rhizoma	C57BL/6J mice	Reduce oxidative stress. Downregulate the expression of Nrf2, HMOX-1, and PTGS2. Increase the expression of GPX4. Inhibit the Nrf2/HMOX-1 signaling pathway.	DN	Inhibit the level of ferroptosis in the treatment of DN.
Germacrone [38]	Rhizoma Curcuma	db/db mice	Regulate ferroptosis by targeting mmu_mmu_circRNA_0000309/miR-188–3p/GPX4 signaling pathway.	DN	Ameliorate kidney damage.
Berberine [85]	Rhizoma Coptidis	MPC5	Activate Nrf2/HO-1/GPX4 pathway.	DN	Alleviate podocyte plasma membrane blistering and mitochondrial shrinkage under high glucose conditions.
Naringin [86]	Citrus plants	SD rats	Regulate ferroptosis by targeting the Nrf2/GPX4 pathway.	DCAN	Relieve DCAN mediated by the P2Y14 receptor of satellite glial cells in the superior cervical ganglia.
Resveratrol [87]	Mainly from grapes, polygonum, peanuts and other plants.	H9c2 cells	Inhibit ferroptosis by up-regulating the expression of HSF1.	DCM	Improve HG-induced cardiomyocyte injury.
Gegen Qinlian Decoction (GQD) [88]	Including Pueraria, Scutellaria, Coptidis, Licorice.	C57BL/KsJ-db/db mice	Regulate the expression of ferroptosis-related genes, reduce the level of lipid peroxidation in myocardial tissue, and up-regulate the expression of GPX4.	DCM	Improve cardiac remodeling and diastolic function in db/db mice with damp-heat syndrome.
Sulforaphane [52]	Cruciferous vegetables like cauliflower.	ECTs AMPKa2-KOmale mice	Activate NRF2 and inhibite cardiac cell ferroptosis by upregulating ferritin and SLC7A11 levels.	DCM	Prevent diabetic cardiomyopathy via AMPK/Nrf2 pathways.
Baicalein [89]	Scutellaria	BMSCs SD rats	Inhibition of ferroptosis by up-regulating the SLC7A11/GPX4 axis.	DOP	Prevent the occurrence of DOP.
Artemisinin [90]	Compositae Artemisia annua	C57BL/6J	Reduce the ROS and MDA contents, increase Nrf2, HO-1, GSH and GPX4.	DCI	Improve cognitive impairment by inhibiting hippocampal ferroptosis via activating Nrf2.
Astragaloside-IV [39]	Astragalus membranaceus	ARPE-19 RPE	Increase mir-138–5p expression in RPE cells and promote expression of Sirt1 and Nrf2 in the nucleus.	DR	Alleviate high- glucose-induced ferroptosis by disrupting the expression of miR-138–5p/Sirt1/Nrf2.
Tu-Xian Mixture [68]	It is composed of dodder, fairy spirit spleen, privet root, pueraria and rhodiola.	SD rat	It may be related to the ferroptosis pathway.	DCI	Improve the cognitive function of diabetic cognitive dysfunction rats.

Partial abbreviation: SD rats: Sprague-Dawley rats; xCT-: the substrate-specific subunit of system Xc-; FBG: fasting blood glucose; ECTs: engineered cardiac tissues; BMSCs: bone marrow mesenchymal stem cells.

### 3.1 Effects on blood glucose and pancreatic islets

Ginseng berry (GB) is the ripe fruit of the medicinal and edible herb Panax ginseng C.A. Meyer, with significant hypoglycemic effects. Ginsenoside is the main hypoglycemic active component of GB. Heyu Ginsenoside extract 5 (GBE-5) has a more significant hypoglycemic effect than other extract components [77]. Using the kyoto encyclopedia of genes and genomes (KEGG) metabolic pathway enrichment analysis, it is found that ferroptosis may be one of the potential metabolic pathways for GBE-5 components to exert hypoglycemic regulation [77]. Moreover, cryptochlorogenic acid (CCA) is an active compound in mulberry leaves, and CCA could inhibit ferroptosis by activating cystine/glutamate transporter (XC-)/GPX4/Nrf2 and inhibiting NCOA4 in diabetes, thereby reducing islet injury in the diabetic model [78]. Except for CCA， another Chinese herbal extract, quercetin, also has indicated the same protective effect of islet function. In the study, the mice with T2DM were treated with quercetin for 4 months, and were observed differences in T2DM mice that were not treated with the drug. The results showed that the glucose tolerance, diabetes symptoms, homeostasis model assessment of insulin resistance (HOMA-IR), and β-cell homeostasis model assessment (HOMA-β) indexes of mice in the quercetin intervention group were basically normalized, and iron ion levels, Mitochondrial atrophy, and other conditions also improved [79]. Ferroptosis can lead to pancreatic β-cell loss and dysfunction, and quercetin may play a beneficial role in type 2 diabetes by inhibiting pancreatic β-cell ferroptosis [79].

### 3.2 Effects on DN

Radix puerariae, a traditional Chinese herbal medication, has been used to treat patients with DN [91]. Puerarin, the active compound of radix puerariae, improved DN through the anti-oxidative effects in the diabetic milieu [91]. A recent study has shown that puerarin attenuates excessive extracellular matrix accumulation in DN by inhibiting glomerular mesangial cells’ ferroptosis [80]. Platycodin D (PD), isolated from the dried root of Platycodon grandiflorum, is a triterpenoid saponin with various pharmacological properties. Another study has also indicated that PD intervention downregulates ACSL4 and TFR1 expression and upregulates GPX4, FTH-1, and SLC7A11 expression in high glucose-induced HK2 cells, suggesting that PD reverses the effects of HG conditions on cellular ferroptosis [81]. It has been previously reported that platycodin D can protect against alloxan-induced liver injury in diabetic mice by regulating Treg/Th17 balance [92]. This is the first report that PD has a protective effect on diabetic nephropathy, possibly through inhibition of GPX4-mediated ferroptosis. Glab is a bioactive component of licorice [93]. Glab has been reported to exert hypoglycemic and protective effects on DM and its complications via anti-inflammation or antioxidative mechanism [94]. Surprisingly, Glab has been shown to repress ferroptosis by increasing SOD and GSH activity, GPX4, SLC7A11, and SLC3A2 expression, and decreasing MDA and iron concentrations, and TFR1 expression, *in vivo* and *in vitro* [82]. It follows that Glab may improve renal function and pathological changes in diabetic nephropathy mice by inhibiting ferroptosis [82]. Calyxine is an isoflavone extracted from the Astragalus root. Astragalus is a traditional medicinal plant widely used in China and has important medicinal value for various diseases such as diabetes [95]. One study has found that calycosin inhibits HG-induced elevation of LDH, MDA, lipid ROS, and NCOA4 in HK2 cells and restores glutathione and GPX4 expression levels, but erastin prevents the appeal effect caused by calycosin [83]. Calycosin may improve renal function in diabetic nephropathy mice by regulating cellular ferroptosis. Sennoside A (SA) the main component of rhubarb, can suppress hyperglycemia and improve complications of type 2 diabetes [96], but the mechanism is not very clear. It has been reported that SA can significantly improve the oxidative stress response of DKD mice, down-regulate the expression of Nrf2, HMOX-1, and PTGS2, and increase the expression of GPX4 [84]. Berberine is an alkaloid extracted from the rhizome of the natural plant Coptis Chinensis, which has anti-inflammatory, anti-oxidative stress, and hypoglycemic effects [97]. Under a high glucose environment, podocytes undergo ferroptosis, and berberine can alleviate this phenomenon, which may be related to the Nrf2/HO-1/GPX4 pathway [85]. As described above， umbelliferone delays the progression of diabetic nephropathy by inhibiting ferroptosis through activation of the Nrf-2/HO-1 pathway [27]. Therefore, molecules on the Nrf2/HO-1/GPX4 pathway may be promising intervention targets for the treatment of diabetic nephropathy. Germacrone is the principal bioactive component of Rhizoma Curcuma, which is discovered to exert a leading impact on many diseases, including anti-inflammation and anti-apoptotic functions [98]. A novel identified circular RNA, mmu_mmu_circRNA_0000309 involves in Germacrone-mediated the improvement of diabetic nephropathy through regulating ferroptosis by targeting miR-188–3p/GPX4 signaling axis [38].

### 3.3 Effects on diabetic cardiovascular disease

Diabetic cardiac autonomic neuropathy (DCAN) is one of the main complications of diabetes, which can cause tachycardia, orthostatic hypotension, silent myocardial ischemia, prolongation of the QT interval, etc. Previous studies have shown that satellite glial cells (SGCs) in the superior cervical ganglion (SCG) play an integral role in the progression of DCAN [99]. Naringin, a traditional Chinese medicine, is an important flavonoid extracted from citrus plants and has various functions such as anti-inflammatory, anti-oxidation, and improving metabolism [100]. A current study has found that naringin can effectively alleviate DCAN, and ferroptosis mediated by the Nrf-2/GPX4 pathway may become one of the main mechanisms for alleviating the progression of DCAN [86]. Diabetic cardiomyopathy (DCM) is a common clinical-specific cardiomyopathy independent of coronary heart disease and hypertension, and is one of the important causes of death in patients with diabetes. A study has shown that resveratrol inhibits ferroptosis and improves high glucose-induced cardiomyocyte injury by up-regulating the expression of HSF1 [87]. Another study has investigated that Gegen Qinlian Decoction can improve cardiac remodeling and diastolic function in diabetic mice with damp-heat, which may be related to the inhibition of myocardial cell ferroptosis [88]. However, the molecular mechanism of Gegen Qinlian Decoction's inhibition of cardiomyocyte ferroptosis is still unclear and needs to be further studied. Furthermore, sulforaphane can inhibit ferroptosis in cardiomyocytes of DCM mice by activating Nrf-2 [52], which suggests that we can properly eat some vegetables rich in these substances to prevent the related diseases caused by cell ferroptosis in our daily life.

### 3.4 Effects on the other complications of diabetes

Oxidative stress secondary to chronic hyperglycemia is an important pathological mechanism of DOP. The effect of baicalein on DOP has been found that it can reduce the level of oxidative stress, inhibit ferroptosis, and reduce bone damage, and its mechanism may be related to the inhibition of SLC7A11/GPX4 axis [89]. This study provides a scientific and experimental basis for the clinical application of baicalein in the prevention and treatment of DOP. Artemisinin, a TCM isolated from the Compositae plant Artemisia annua, can penetrate the blood-brain barrier [101] and can increase insulin secretion and sensitivity in T2DM mice [69]. In addition, artemisinin also inhibits hippocampal ferroptosis by activating Nrf2 to improve cognitive dysfunction in mice with type 2 diabetes [90]. Astragaloside-IV (AS-IV) (C41H68O14) is a high-purity natural product extracted from Astragalus membranaceus. It has been reported that AS-IV can increase Sirt1/Nrf2 activity and cellular antioxidant capacity by inhibiting the expression of miR-138–5p to inhibit ferroptosis and thereby reduce cell death, which may inhibit the pathological process of DR [39]. Paeoniflorin is the main active component of Paeonia lactiflora, with antioxidant, anti-inflammatory, and other biological functions [102]. Diabetic foot ulcer is one of the most common complications in diabetic patients, leading to limb ischemia and even amputation [103]. A study by Xiaolong Sun et al. has shown that paeoniflorin can play an active role in diabetic wound healing through the Nrf2/HO-1 pathway [104]. It is speculated that paeoniflorin may inhibit ferroptosis through the Nrf2/HO-1 pathway to improve wound healing of diabetic foot. In addition, puerarin inhibits oxidative stress through the Nrf2/HO-1 signaling pathway, thereby preventing the development and progression of cataracts in diabetic rats [105]. Whether puerarin participates in the regulation of iron metabolism and ferroptosis to delay the occurrence and development of cataract in diabetic rats needs further research to confirm.

## 4 Conclusions and prospects

In conclusion, ferroptosis is closely related to the occurrence and development of diabetes and its complications. In an environment of high glucose, diabetic patients may lead to the occurrence of ferroptosis in tissues and organs through iron metabolism, GPX4 metabolism, lipid metabolism, and other pathways, thereby further causing the development of diabetes and its complications. Ferroptosis is affected by a variety of metabolic factors, and the detailed molecular mechanisms of diabetes and its complications need further study. The studies described herein have found that herbs or herbal natural compounds can improve diabetes and its complications through the ferroptosis pathway. It is not difficult to find that most of the herbs or herbal natural compounds described in the review have shown anti-inflammatory and anti-oxidative stress effects in previous studies. This suggests that there may be more herbal medicines or herbal natural compounds with anti-inflammatory and anti-oxidative stress that may ameliorate diabetes and its complications in part through the ferroptosis pathway. This provides a more solid theoretical basis for the treatment of diabetic complications with traditional Chinese medicine. In addition, extracting the natural active components of some herbs may become a promising targeted drug for improving diabetes and its complications. However, the current research in this field is still shallow, limited to cell experiments and animal experiments, and the molecular mechanism is not yet in-depth. In the future, specific binding between these herbs or herbal natural compounds and molecules on the ferroptosis pathway may be a research direction. The application of modern technical methods such as epigenomics and bioinformatics may help to study this field more systematically and in-depth.

## Ethics statement

Review and/or approval by an ethics committee was not needed for this review, and informed consent was not required for this review, because it does not involve any clinical trials or animal experiments, and the opinions expressed in the review were some viewpoints and hypotheses based on previous experiments.

## Data availability statement

No data was used for the research described in the article.

## CRediT authorship contribution statement

**Cuiping Liu:** Writing – review & editing, Writing – original draft. **Wuxi Wang:** Writing – review & editing. **Junling Gu:** Writing – review & editing, Writing – original draft, Supervision, Funding acquisition.

## Declaration of competing interest

The authors declare that they have no known competing financial interests or personal relationships that could have appeared to influence the work reported in this paper.
